# Bevacizumab-induced esophageal pleural fistula during maintenance therapy without radiation in lung cancer

**DOI:** 10.1186/s12890-021-01750-w

**Published:** 2021-11-25

**Authors:** Ting Wang, Asmitananda Thakur, Baoqing Chen

**Affiliations:** 1 Department of Respiratory Medicine, Xi’an People’s Hospital (Xi’an No.4 Hospital), No.21 Jiefang Road, Xi’an, 710004 China; 2 Chest Clinic, NATA, Morang, Biratnagar, Nepal

**Keywords:** Bevacizumab, Lung cancer, Esophageal pleural fistula, Case report

## Abstract

**Background:**

Esophageal pleural fistula (EPF) is a rare but fatal complication associated with bevacizumab use; however, cases reports of EPF caused by bevacizumab have not been previously published.

**Case presentation:**

A 66-year-old male patient diagnosed with stage IV lung adenocarcinoma on April 24, 2020 received 6 cycles of platinum-containing dual chemotherapy combined with bevacizumab followed by three cycles of bevacizumab monotherapy. Five days before admission, he experienced chest tightness, dyspnea, and right chest pain. Bed-side X-ray examination revealed a massive right hydrothorax, and food was found in the extracted pleural effusion. EPF was further confirmed by upper gastrointestinal radiography after oral administration of iohexol. The patient underwent jejunostomy as the distal esophagus could not be identified on gastroscopy, and eventually died of septic shock on January 16, 2021.

**Conclusions:**

It is necessary to pay attention to EPF during bevacizumab use in patients with or without risk factors.

## Background

Bevacizumab, the first antiangiogenic drug approved for lung cancer, has been recommended by the National Comprehensive Cancer Network guidelines as a first-line treatment for advanced metastatic non-small-cell lung cancer, as well as a monotherapy after chemotherapy [1]. Bevacizumab reduces angiogenesis in neoplastic tissue by blocking the binding between vascular endothelial growth factor and its receptor. In addition, it increases the concentration of chemotherapeutic drugs by strengthening vasopermeability, thus enhancing their bioavailability [2]. The main adverse effects of bevacizumab are hypertension and bleeding, while gastrointestinal perforation and visceral fistula are uncommon, and esophageal pleural fistula (EPF) is even rarer [3]. In a literature review, we identified 11 cases of bevacizumab-induced tracheoesophageal fistula (TEF), but found no related reports of EPF caused by bevacizumab [4, 5]. Here, we retrospectively analyzed the clinical data of this rare case and reviewed the related literature.

## Case presentation

**Fig. 1:**
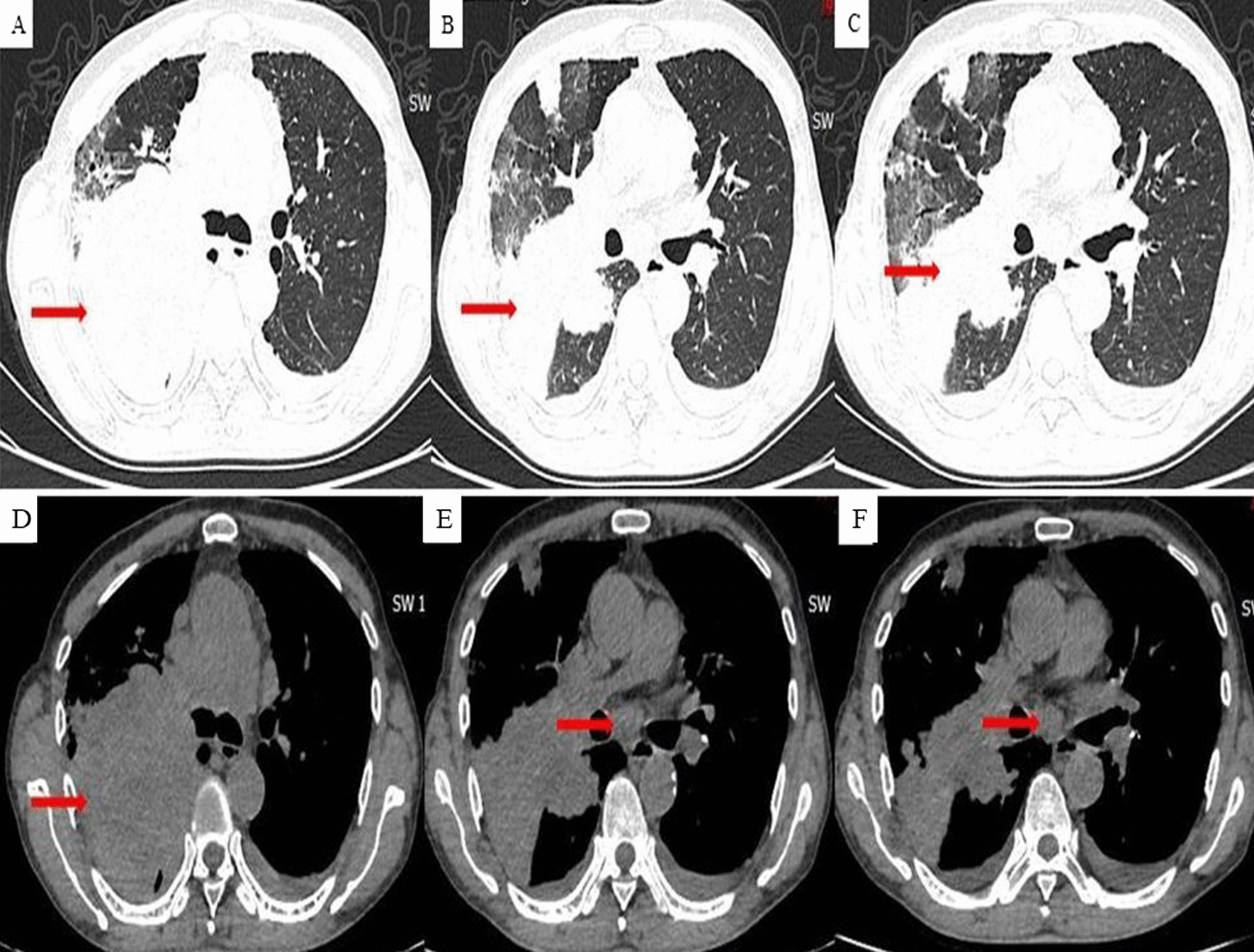
Chest CT image at the initial visit. A mass and mediastinal enlarged lymph node (red arrow) could be seen in the upper lobe of the right lung. **a** Pulmonary window, trachea bifurcation level, **b** and **c** pulmonary window, left upper lobe bronchial branch level. **d** Mediastinal window, trachea bifurcation level. **e** and **f** Mediastinal window, left upper lobe bronchial branch level

**Fig. 2:**
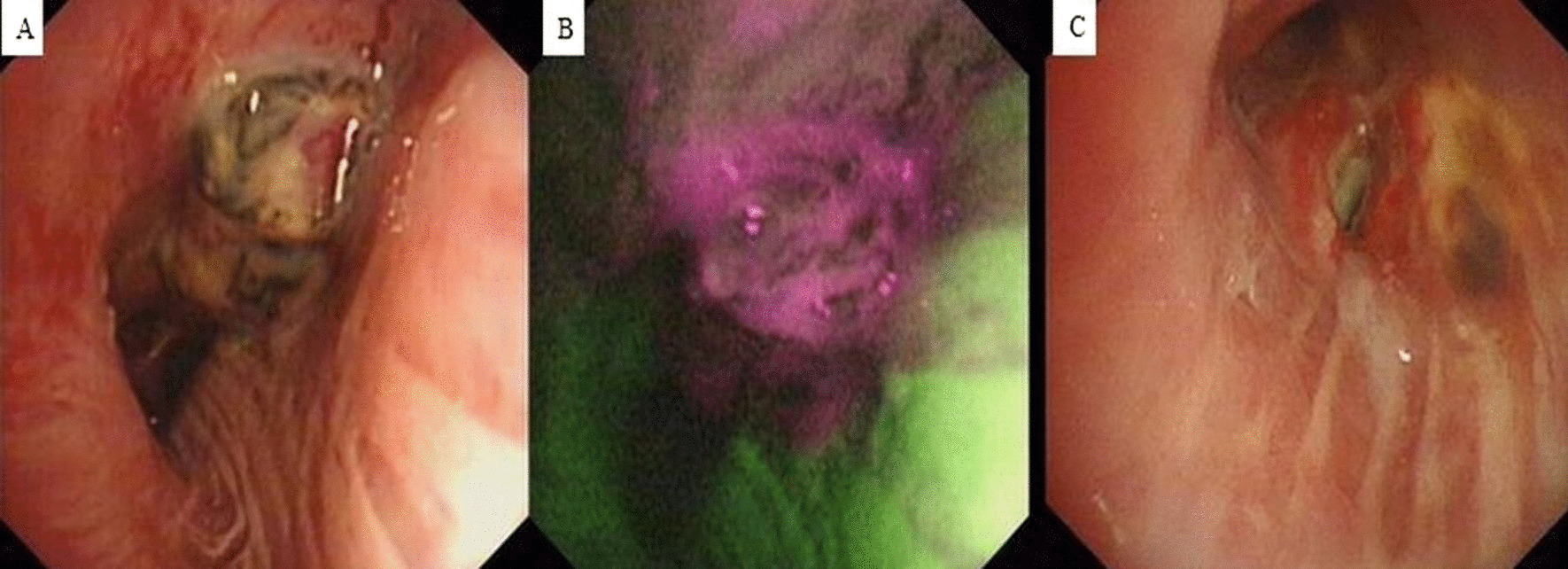
**a** and **b** A bronchial neoplasm with discoloration under fluorescence could be seen in the upper lobe of right lung by bronchoscopy. **c** The tumor decreased after 4 months of treatment under bronchoscopy

A 66-year-old man with a long history of smoking (20 cigarettes a day for more than 30 years) was admitted to the respiratory department of our hospital on April 16, 2020, with a history of right chest pain over the past 10 days. Chest computed tomography revealed a space-occupying lesion in the right upper lobe and multiple mediastinal lymph node enlargement (Fig. 1a–f). The patient underwent bronchoscopy, and a bronchial neoplasm with discoloration under fluorescence was observed in the upper lobe of the right lung (Fig. 2a, b). The histopathological diagnosis was moderately differentiated adenocarcinoma. Genetic testing revealed no mutant genes, including epidermal growth factor receptor-21 (EGFR-21) and echinoderm microtubule-associated protein-like 4-anaplastic lymphoma kinase (EMLA4-ALK) fusion.

The patient was diagnosed with lung adenocarcinoma (T_4_N_2_M_1_), according to the Union for International Cancer Control 7th edition TNM classification of lung cancer. Other detailed laboratory data were as follows: carcinoembryonic antigen = 172 ng/ml (normal range: 0-5 ng/ml), cytokeratin 21–1 fragment = 37.7 ng/ml (normal range: 0–3.3 ng/ml), neuron-specific enolase = 21.00 ng/ml (normal range: 0–16.3 ng/ml). His Eastern Cooperative Oncology Group Performance Status score was 0. The patient was treated with six cycles of systemic chemotherapy combined with bevacizumab, specifically; pemetrexed 800 mg/d + cisplatin 40 mg/d1 − 3 + bevacizumab 300 mg/d, followed by 3 cycles of bevacizumab monotherapy for every 21 days (300 mg/d).Imaging every two months revealed that the tumor decreased in size (Fig. 2c), and serum lung cancer biomarkers were significantly reduced.

**Fig. 3:**
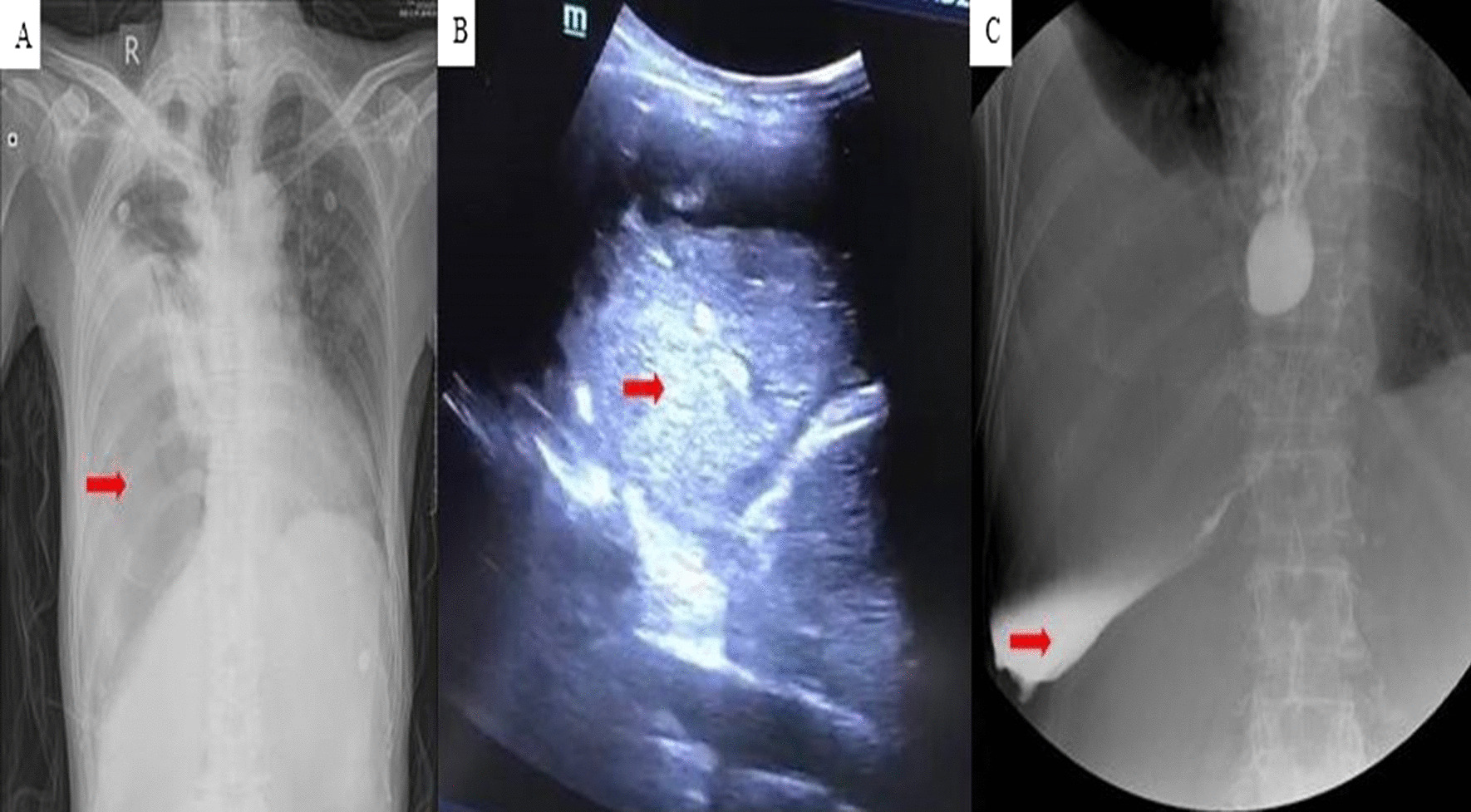
**a** Chest X-ray demonstrated a massive right hydrothorax (red arrow). **b** Chest ultrasound showed a floating hyper echoic mass (red arrow). **c** Upper gastrointestinal radiography gave us information that contrast agent was found in the chest cavity (red arrow)

**Fig. 4:**
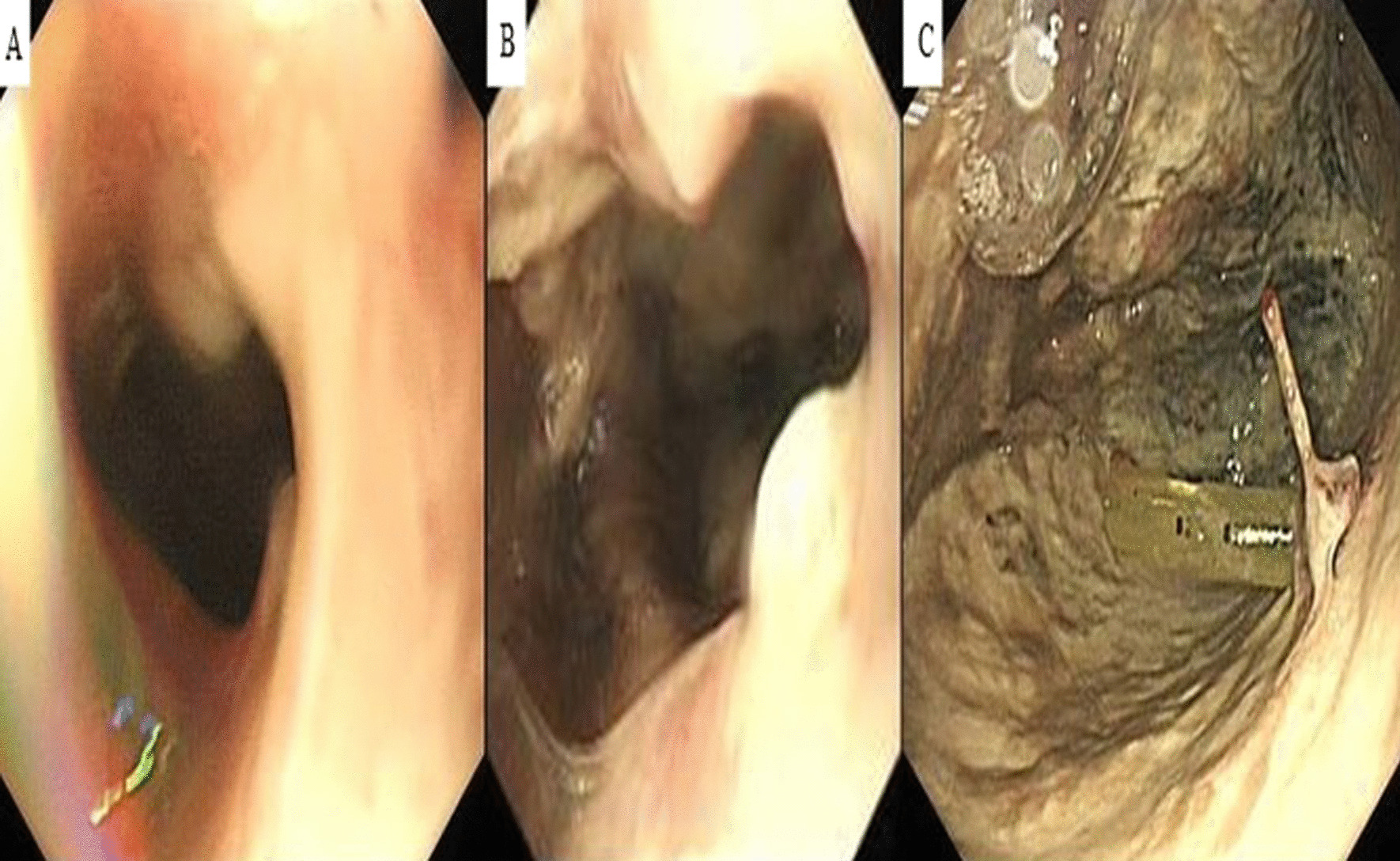
Esophagoscope images. **a** Esophageal orifice, **b** esophageal fistula, **c** intrathoracic drainage tube

On November 24, 2020, the patient was admitted to our department again with chest tightness and shortness of breath for five days. He complained of severe shortness of breath when eating, as well as right chest pain. His temperature was 36.8 °C, respiratory rate was 25 breaths/min, and the respiratory sound of the right lower lung disappeared. Laboratory data included a white blood cell count of 16.35*10^9^ /L, neutrophil percentage 89.1%, C-reactive protein 310.7 mg/l, procalcitonin 2.548 ng/ml, and D-dimer 4.60ug/ml. All lung cancer biomarkers were normal. Bed-side chest radiography revealed a severe right hydrothorax (Fig. 3a). Ultrasound detected an unclear fluid sonolucent area in the right thorax, with a floating hyperechoic mass (Fig. 3b). We performed thoracocentesis, which resulted in the extraction of 50 ml melicera, gray pleural effusion, with food (egg yolk) inside. The patient was subsequently treated with carbapenem antibiotic. EPF was confirmed by upper gastrointestinal radiography after oral administration of iohexol (Fig. 3c). After pleural effusion drainage, the patient underwent gastroscopy, revealing a 15 mm diameter fistulaon the esophagus 30 mm away from the incisor; however, the proximal esophagus of the fistula could not be exposed and the distal esophagus could not be found (Fig. 4a–c). Therefore, we contacted the department of general surgery for consultation and performed jejunostomy for internal nutrition. Unfortunately, the patient died of septic shock on January 16, 2021.

## Discussion and conclusions

**Fig. 5:**
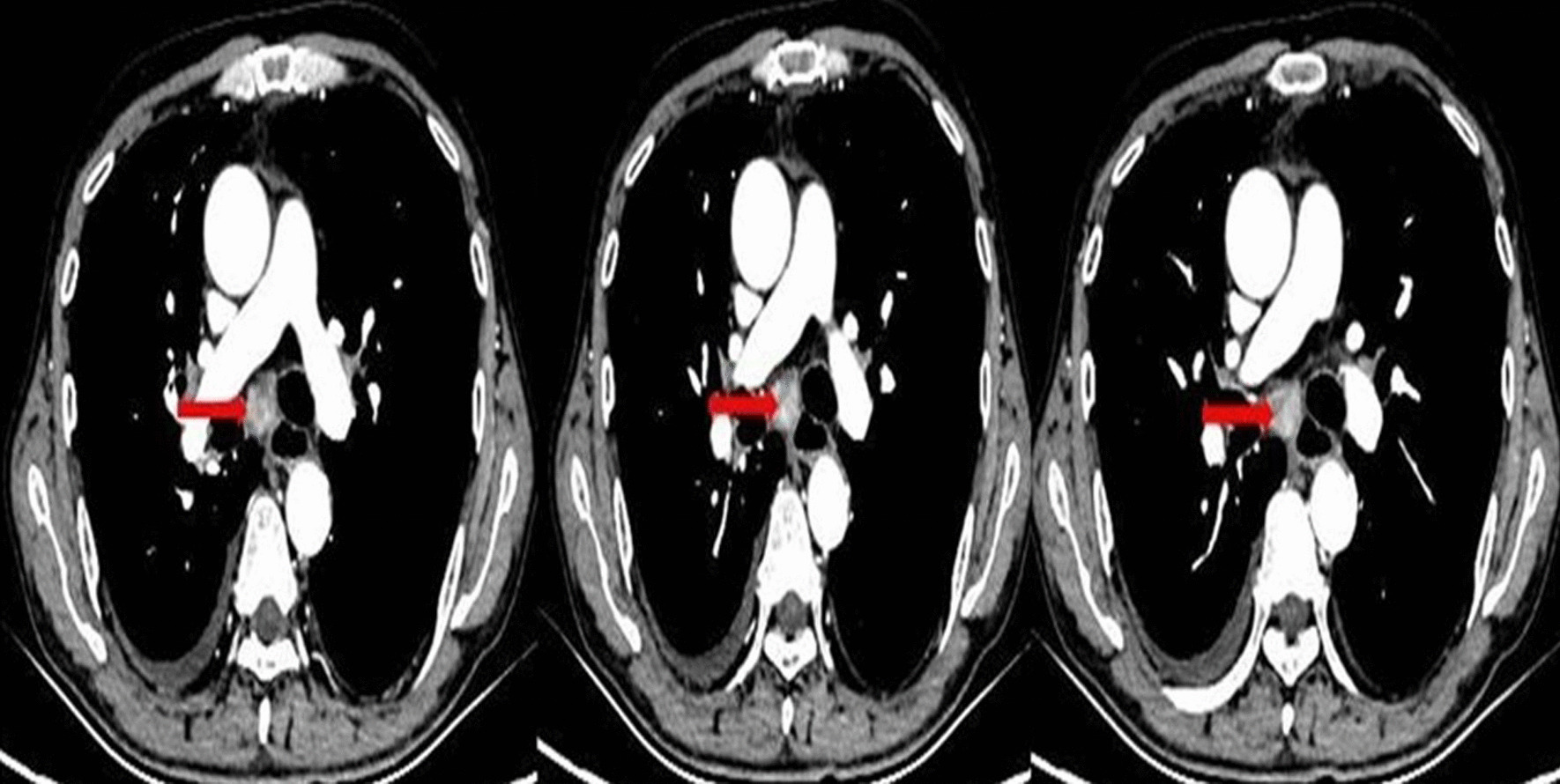
Enhanced CT revealed enlargement of the paraesophageal lymph nodes

By searching the PubMed database, Google Scholar, MEDLINE, Cochrane library, and China National Knowledge Infrastructure for the key words "bevacizumab, esophageal fistula”, a total of 11 cases of TEF caused by bevacizumab were retrieved, including 7 men and 3 women aged 28–67 years, as shown in Table 1 [5]. These reports indicate that the use of bevacizumab can lead to TEF, mainly in patients with related risk factors, such as thoracic radiotherapy or esophageal mucosal lesions. Among these 11 patients, 10 had a history of chest radiotherapy, and 6 had esophageal mucosal lesions, such as Barrett's esophagus and esophagitis caused by chemotherapy. Similar to our case, Kenichi et al. reported that a patient with TEF caused by the use of bevacizumab in 2018 had no history of chest radiotherapy or esophageal disease. The reason for EPF formation was unclear; however, we speculate that the enlarged lymph nodes between the esophagus and pleura may be involved in the formation of the fistula (Fig. 5), and bevacizumab accelerated this formation.

**Table 1:** Previously reported cases on the correlation between TEF and bevacizumab

Article	Age, sex	Tumor type	Treatment	Risk factors	Using time and dosage of Bev	Outcome
Goodgame 2008	28, M	NSCLC	PTX + CBDCA + Bev → Bev	TRT	2 Cycles, 21 days per 1 cycle; 15 mg/kg	NA
Gore 2009	48, M	NSCLC	GEM + CBDCA + Bev → Bev	TRT	1 Cycle; NA	NA
Spigel 2010	62, F	NSCLC	PEM + CBDCA + Bev	TRT/esophagitis	8 Cycles, 21 days per 1 cycle; 15 mg/kg	NA
	55, M	NSCLC	PEM + CBDCA + Bev	TRT/esophagitis	10 Cycles, 21 days per 1 cycle; 15 mg/kg	NA
	57, F	SCLC	CPT-11 + CBDCA + Bev	TRT/esophagitis	16 Cycles, 14 days per 1 cycle; 10 mg/kg	NA
	57, F	SCLC	CPT-11 + CBDCA + Bev	TRT/esophagitis	10 Cycles, 14 days per 1 cycle; 10 mg/kg	Dead due to TEF
	67, M	SCLC	CPT-11 + CBDCA + Bev	TRT/esophagitis	NA	NA
Socinski 2012	NA	NSCLC	PEM + CBDCA + Bev	TRT	NA	NA
Schreiber 2012	40, M	NSCLC	PEM + CBDCA + Bev	TRT/Barret’s esophagus	1 Cycle; 7.5 mg/m^2^	Dead due to hemoptysis
Kenichi 2018	66, M	NSCLC	PEM + CBDCA + Bev	None	1 Cycle; 15 mg/kg	Dead due to lung cancer
Zhang 2020	54, M	NSCLC	PEM + CBDCA + Bev	TRT	4 Cycles, 21 days for 1 cycle; 7.5 mg/kg	NA

*NSCLC* non-small-cell lung cancer, *SCLC* small-cell lung cancer, *TEF* tracheoesophageal fistula, *TRT* thoracic radiation therapy, *CDDP* cisplatin, *CBDCA* carboplatin, *ETP* etoposide, *PTX* paclitaxel, *Bev* bevacizumab, *CPT-11* Irinotecan, *PEM* pemetrexed, *GEM* gemcitabine, *NA* no available

The esophagus is the only organ in the digestive tract that lacks a serosa. Compared with the upper and middle segments, the lower esophagus has fewer adjacent organs and is relatively free from the pleura. Therefore, when the intra-abdominal pressure rises suddenly, the lower segment of the esophagus is more likely to rupture, resulting in EPF. Unlike its counterpart, the right esophagus is closely attached to the pleura, has no surrounding soft tissues, and is prone to esophageal fistula [6]. After the occurrence of EPF, gastric contents enter the chest through the fistula, causing infection and inflammation. Due to the presence of atypical symptoms at an early stage, EPF is easily misdiagnosed as pulmonary embolism, pneumothorax, or pneumonia, and thus has to be characterized by the discovery of gastric contents in the pleural effusion. Patients may experience chest pain and different degrees of dyspnea due to the stimulation of gastric acid and compression caused by effusion. In such cases, the patient may experience aggravated chest tightness, shortness of breath, andorthopnea, which are characteristic of EPF [7].

EPF is a disease with poor prognosis, with more than 80% mortality if treated conservatively. Common therapeutic regimens include micro-invasive treatment guided by endoscopy, stent implantation, and surgical treatment [8]. Stent implantation has a high success rate and is the most commonly used treatment. According to the report of Kim et al. [9], 8 of 9 EPF patients treated with recyclable self-expanding metal covered stents were successfully implanted, result in a significantly improved prognosis. For previously reported TEF cases induced by bevacizumab, clinicians adopted esophageal stent placement under endoscopy, and all the patients’ symptoms improved. However, in this case, the patient could not benefit from these treatments due to severe infection, and soon died of septic shock.

In conclusion, esophageal pleura fistula is a rare but fatal complication associated with the use of bevacizumab. Thus, it is necessary to be vigilant of the possibility of EPF in patients treated with bevacizumab whether or not the patient has risk factors.

## Data Availability

All the data supporting our findings is contained within the manuscript. The patient’s gene sequences tested have been uploaded to NCBI database and are available with the follow link: https://www.ncbi.nlm.nih.gov/sra/PRJNA780355.

## Abbreviations

EPF: Esophageal pleural fistula
TEF: Tracheoesophageal fistula
EGFR-21: Epidermal growth factor receptor-21
EMLA4-ALK: Echinoderm microtubule-associated protein-like 4-anaplastic lymphoma kinase
SCLC: Small-cell lung cancer
TRT: Thoracic radiation therapy
CDDP: Cisplatin
CBDCA: Carboplatin
ETP: Etoposide
PTX: Paclitaxel
Bev: Bevacizumab
CPT-11: Irinotecan
PEM: Pemetrexed
GEM: Gemcitabine
NA: No available
